# Comparison of contraceptive failures associated with CYP3A4-inducing drug-drug interactions by route of hormonal contraceptive in an adverse event reporting system

**DOI:** 10.1016/j.contraception.2020.12.002

**Published:** 2021-04

**Authors:** Tomiko Sunaga, Brian Cicali, Stephan Schmidt, Joshua Brown

**Affiliations:** a Center for Drug Evaluation and Safety, Department of Pharmaceutical Outcomes and Policy, University of Florida College of Pharmacy, Gainesville, FL, United States; b Showa University, School of Pharmacy, Tokyo, Japan; c Center for Pharmacometrics and Systems Pharmacology, Department of Pharmaceutics, University of Florida College of Pharmacy, Orlando, FL, United States

**Keywords:** Hormonal contraception, Drug-drug interaction, CYP3A4, Inducers, Unintended pregnancy, Contraceptive failure

## Abstract

**Objective:**

To estimate associations between contraceptive failures and concomitant CYP3A4-inducing medications by route of administration.

**Study design:**

Comparison of unintended pregnancy outcomes within U.S. Food and Drug Administration's Adverse Event Reporting System by couse of CYP3A4-inducing drugs and route of administration for levonorgestrel and etonogestrel/desogestrel.

**Results:**

Among 14,504 levonorgestrel case reports, the reporting odds ratio (ROR) was increased for oral (ROR = 4.2 [3.0–5.7]), implants (ROR = 8.0 [5.8–11.0]), but not intrauterine (ROR = 0.9 [0.6–1.3]) levonorgestrel products. For 9348 etonogestrel/desogestrel case reports, oral and vaginal products were not associated with contraceptive failure. Etonogestrel containing implants (ROR = 4.9 [4.1–5.9]) were associated with increased contraceptive failure.

**Conclusion:**

Levonorgestrel containing combination oral products and implants containing levonorgestrel or etonogestrel were prone to CYP3A4-inducing drug-drug interactions that may increase contraceptive failures.

**Implications:**

The progestin components of hormonal contraceptives are susceptible to drug-drug interactions, but this susceptibility is influenced by route of administration. This study provides evidence from an Adverse Event Reporting System that CYP3A4-inducing medications increase the risk of unintended pregnancy for oral and implant contraceptives but not intrauterine or vaginal devices.

## 1 Introduction

An estimated 44% of all pregnancies worldwide are unintended and half occur among users of contraceptive methods [1]. Contraception is used by nearly 90% of women at risk for unintended pregnancy and consists of mostly oral (68.8% of contraceptive users), 15% long-acting intrauterine or implant devices, and 16.3% other routes such as patches, rings, or injectables [1]. Contraceptive failure rates vary considerably by administration routes, for example, 7% to 9% for oral products and near or less than 1% for implant and intrauterine devices [2].

Growing evidence suggests a role for concomitant use of other medications and potential drug-drug interactions with hormonal agents [3]. The progestin component provides the major contraceptive effect [4]; thus, drug-drug interactions mediated through cytochrome-P450 (CYP) 3A4 may be important [5]. CYP3A4 drug-drug interaction warnings are part of the Food and Drug Administration's labeling guidance [6] for all hormonal contraceptive products and Centers for Disease Control & Prevention [2] and World Health Organization [7] guidance provide recommendations for contraceptive products in the presence of drug interactions.

The objective of this study was to evaluate case reports of contraceptive failure (i.e., unintended pregnancy) among users of levonorgestrel-containing oral, intrauterine, and implant products as well as etonogestrel-containing implants, vaginal rings, and oral (desogestrel pro-drug) products exposed to CYP3A4-inducing medications. It was hypothesized that due to lower systemic exposure, intrauterine and vaginal devices would not be impact by the drug-drug interaction while oral and implants would be strongly impacted.

## 2 Methods

Case reports from the U.S. Food and Drug Administration's Adverse Event Reporting System (FAERS) from the years 1971 through Q1/2020 were used. A structured query captured all FAERS case reports of levonorgestrel and etonogestrel/desogestrel products including all combined orals (with ethinyl estradiol), intrauterine or intravaginal devices, and implants. Levonorgestrel and etonogestrel/desogestrel were specifically selected as they are available in a variety of routes of administration compared to other progestins. These case reports were stratified to those that reported an outcome of “unintended pregnancy” versus all other reactions based on Medical Dictionary for Regulatory Activities (MedDRA) definitions. CYP3A4-inducing medications were defined based on DrugBank (drugbank.ca), and identified on the same case report as a binary exposure (yes/no; Table 1).

**Table 1:** CYP3A4-inducing medications captured on U.S. Food and Drug Administration Adverse Event Reporting System case reports for contraceptive products containing levonorgestrel or etonogestrel/desogestrel

Concomitant inducer drugs	Levonorgestrel			Etonogestrel		
	**Oral**	**Implant**	**Intrauterine**	**Implant**	**Vaginal ring**	**Oral**
	***n* (%)**	***n* (%)**	***n* (%)**	***n* (%)**	***n* (%)**	***n* (%)**
Carbamazepine	54 (1.4%)	67 (2.7%)	52 (0.6%)	84 (1.8%)	7 (0.2%)	8 (0.7%)
Phenytoin	26 (0.7%)	89 (3.6%)	11 (0.1%)	12 (0.3%)	7 (0.2%)	3 (0.3%)
Rifampin	9 (0.2%)	10 (0.4%)	0 (0%)	40 (0.8%)	2 (0.1%)	3 (0.3%)
Enzalutamide	0 (0%)	0 (0%)	0 (0%)	1 (0%)	0 (0%)	0 (0%)
St. John's Wort	2 (0.1%)	0 (0%)	4 (0%)	5 (0.1%)	2 (0.1%)	0 (0%)
Phenobarbital	13 (0.3%)	21 (0.8%)	6 (0.1%)	7 (0.1%)	0 (0%)	1 (0.1%)
Dexamethasone	8 (0.2%)	8 (0.3%)	17 (0.2%)	0 (0%)	4 (0.1%)	6 (0.5%)
Pentobarbital	0 (0%)	0 (0%)	1 (0%)	0 (0%)	0 (0%)	0 (0%)
Lumacaftor	0 (0%)	0 (0%)	1 (0%)	0 (0%)	0 (0%)	1 (0.1%)
Primidone	3 (0.1%)	6 (0.2%)	2 (0%)	1 (0%)	1 (0%)	0 (0%)
Bosentan	3 (0.1%)	0 (0%)	1 (0%)	0 (0%)	1 (0%)	1 (0.1%)
Efavirenz	1 (0%)	0 (0%)	0 (0%)	73 (1.5%)	0 (0%)	0 (0%)
Modafanil	11 (0.3%)	0 (0%)	23 (0.3%)	1 (0%)	9 (0.3%)	1 (0.1%)
Armodafinil	4 (0.1%)	0 (0%)	13 (0.2%)	4 (0.1%)	1 (0%)	0 (0%)
Topiramate	62 (1.6%)	0 (0%)	188 (2.3%)	108 (2.3%)	141 (4%)	11 (1%)
Butalbital	3 (0.1%)	1 (0%)	12 (0.1%)	6 (0.1%)	4 (0.1%)	0 (0%)
Pioglitazone	0 (0%)	0 (0%)	2 (0%)	3 (0.1%)	2 (0.1%)	0 (0%)
Oxcarbazepine	9 (0.2%)	0 (0%)	26 (0.3%)	31 (0.7%)	13 (0.4%)	0 (0%)

*n*, the number of case reports.

We calculated reporting odds ratios (ROR) and 95% confidence intervals from the 2 × 2 exposure-outcome contingency tables to measure disproportionality of unintended pregnancy case reports in exposed versus unexposed groups. A common interpretation of a significant effect is based on the ROR's lower 95% confidence limit of ≥2.0. To control for demographic differences associated with choice of contraceptive method, we stratified by contraceptive route and made the comparison those exposed versus those unexposed to a CYP3A4 inducer. Analyses were conducted using SAS version 9.4 (SAS Institute, Cary, NC).

## 3 Results

A total of 14,504 levonorgestrel and 9348 etonogestrel/desogestrel case reports were identified and 684 (4.7%) and 864 (9.2%) involved a CYP3A4 inducer (Table 2). Among oral levonorgestrel products, there was a significant association with CYP3A4 inducers and unintended pregnancy (ROR = 4.2 [3.0–5.7]) but not for oral desogestrel. Use of CYP3A4 inducers with intrauterine levonorgestrel was neither associated with unintended pregnancies (ROR = 0.9 [0.6–1.3], *p* = 0.52) nor for etonogestrel vaginal rings (ROR = 1.3 [0.8–2.0]). Case reports among contraceptive implants showed a significant association with CYP3A4 inducers and levonorgestrel and etonogestrel, respectively (ROR = 8.0 [5.8–11.0] and ROR = 4.9 [4.1–5.9]).

**Table 2:** Prevalence of case reports, use of CYP3A4-inducing medications, and reporting odds ratios (ROR) and 95% confidence intervals for the association between contraceptive failures (unintended pregnancy) and concomitant medication use by route of administration with levonorgestrel and etonogestrel/desogestrel containing products

Contraceptive product	Unintended pregnancy case reports, *n* (%)	All other case reports, *n* (%)	ROR (95% CI)
Ethinyl estradiol/levonorgestrel oral			
CYP3A4 inducer drugs group	63 (32.8)	129 (67.2)	4.2 (3.0–5.7)
Other drugs group	377 (10.5)	3222 (89.5)	
Levonorgestrel intrauterine device			
CYP3A4 inducer drugs group	32 (10.3)	279 (89.7)	0.9 (0.6–1.3)
Other drugs group	908 (11.5)	7013 (88.5)	
Levonorgestrel implant			
CYP3A4 inducer drugs group	94 (51.9)	87 (48.1)	8.0 (5.8–11.0)
Other drugs group	273 (11.9)	2027 (88.1)	
Ethinyl estradiol/desogestrel (pro-drug) oral			
CYP3A4 inducer drugs group	4 (11.8)	30 (88.2)	0.6 (0.2–1.8)
Other drugs group	185 (17.4)	878 (82.6)	
Etonogestrel vaginal ring			
CYP3A4 inducer drugs group	21 (10.9)	172 (89.1)	1.3 (0.8–2.0)
Other drugs group	291 (8.7)	3044 (91.3)	
Etonogestrel implant			
CYP3A4 inducer drugs group	271 (42.5)	366 (57.5)	4.9 (4.0–5.9)
Other drugs group	536 (13.1)	3550 (86.9)	

*n*, the number of case reports; ROR, reporting odds ratio; CI, 95% confidence interval.

## 4 Discussion

These results indicate a differential association between use of CYP3A4-inducing medications and unintended pregnancies among hormonal contraceptive users dependent on route of administration. Oral and implant contraceptive products were susceptible to these drug-drug interactions but neither intrauterine nor vaginal ring products. These results are clinically important as both intrauterine and implant devices are long-acting and reversible options with much lower failure rates with typical use compared to oral contraception [8]. However, our results indicate that in the presence of metabolic inducers that increase the clearance of hormonal agents, intrauterine or vaginal devices may be preferred.

The finding that the levonorgestrel implant had a higher ROR compared to oral products is noteworthy. While in typical use implants have favorable failure rates [8], they have lower systemic exposure compared to orals, which explains the observed susceptibility. Mean blood levels of levonorgestrel with implants range 300 to 400 ng/L with declining exposure with longer indwell time while oral products have mean concentrations between 1370 and 2050 ng/L [9]. Similar effects have been observed with the newer generation etonogestrel-containing implant, which also had a significant signal for contraceptive failure in this study [10].

Antiepileptics, especially topiramate, made up the majority of CYP3A4 inducers in this study and may be most relevant in developed nations whereas antiretrovirals and rifamycin antibiotics may be more relevant in low-resource countries. Awareness is needed among various medical specialties in both developed and low resource nations to select appropriate contraception in the presence of drug-drug interactions. These results support recommendations by the Centers for Disease Control and Prevention and World Health Organization for use of intrauterine devices in the presence of drug-drug interactions [2,7].

There are several limitations to our study. FAERS case reports do not include a denominator of medication users and may not be used to report rates between groups. FAERS data are spontaneously reported and subject to reporting biases associated with public knowledge of safety issues, release of new medications, or masking effects due to imbalance in event reporting. Reported reactions or medications reported on case reports are not further adjudicated. To reduce potential biases, we reported results within contraceptive routes of administration and restricted to products containing levonorgestrel or etonogestrel/desogestrel. Other important factors such as body mass index, race, socioeconomic, among others, are not routinely captured in FAERS and may influence contraceptive effectiveness.

In conclusion, in this analysis of FAERS data, we observed disproportionate case reports of unintended pregnancies among patients treated with oral and implant contraceptives when exposed to CYP3A4-inducing drugs. Intrauterine and vaginal ring devices were not impacted by this drug-drug interaction. These findings suggest that intrauterine and vaginal ring contraceptive products may be preferred among women concomitantly using CYP3A4-inducing medications compared to oral or implant contraceptive products. While these results are not confirmatory, future studies should evaluate differences in drug-drug interaction susceptibility by contraceptive routes and progestin components [3].

## Declaration of Competing Interest

The authors declare no conflict of interest.

## Funding

This work was supported by the Bill & Melinda Gates Foundation, Seattle, WA (OPP1185454).

## Appendix A Supplementary materials

Supplementary Data S1 Supplementary Raw Research Data. This is open data under the CC BY license http://creativecommons.org/licenses/by/4.0/ Supplementary Data S1

## References

[1] Kavanaugh ML, Jerman J (2018). Contraceptive method use in the United States: trends and characteristics between 2008, 2012 and 2014. Contraception.

[2] Curtis KM, Tepper NK, Jatlaoui TC, Berry-Bibee E, Horton LG, Zapata LB (2016). U.S. medical eligibility criteria for contraceptive use, 2016. MMWR Recomm Rep.

[3] Lesko LJ, Vozmediano V, Brown JD, Winterstein A, Zhao P, Lippert J (2018). Establishing a multidisciplinary framework to study drug-drug interactions of hormonal contraceptives: an invitation to collaborate. CPT Pharmacometrics Syst Pharmacol.

[4] Erkkola R, Landgren BM (2005). Role of progestins in contraception. Acta Obstet Gynecol Scand.

[5] Johannessen SI, Landmark CJ (2010). Antiepileptic drug interactions - principles and clinical implications. Curr Neuropharmacol.

[6] Akbar M, Berry-Bibee E, Blithe DL, Day RS, Edelman A, Höchel J (2018). FDA public meeting report on "Drug Interactions With Hormonal Contraceptives: Public Health and Drug Development Implications". J Clin Pharmacol.

[7] Medical eligibility criteria for contraceptive use. World Helath Organization. 2015. https://www.who.int/publications/i/item/9789241549158. Accessed November 11, 2020. 26447268

[8] Polis CB, Bradley SE, Bankole A, Onda T, Croft T, Singh S (2016). Typical-use contraceptive failure rates in 43 countries with demographic and health survey data: summary of a detailed report. Contraception.

[9] Reinecke I, Hofmann B, Mesic E, Drenth HJ, Garmann D. (2018). An integrated population pharmacokinetic analysis to characterize levonorgestrel pharmacokinetics after different administration routes. J Clin Pharmacol.

[10] Lazorwitz A, Davis A, Swartz M, Guiahi M. (2017). The effect of carbamazepine on etonogestrel concentrations in contraceptive implant users. Contraception.

